# Lipid Droplets Protect Aging Mitochondria and Thus Promote Lifespan in Yeast Cells

**DOI:** 10.3389/fcell.2021.774985

**Published:** 2021-11-19

**Authors:** Melanie Kovacs, Florian Geltinger, Thomas Verwanger, Richard Weiss, Klaus Richter, Mark Rinnerthaler

**Affiliations:** Department of Biosciences, Paris-Lodron University Salzburg, Salzburg, Austria

**Keywords:** aging, lipid droplet (LD), protein homeostais, mitochondrial damage, ROS-, reactive oxygen species, detoxification

## Abstract

Besides their role as a storage for neutral lipids and sterols, there is increasing evidence that lipid droplets (LDs) are involved in cellular detoxification. LDs are in close contact to a broad variety of organelles where protein- and lipid exchange is mediated. Mitochondria as a main driver of the aging process produce reactive oxygen species (ROS), which damage several cellular components. LDs as highly dynamic organelles mediate a potent detoxification mechanism by taking up toxic lipids and proteins. A stimulation of LDs induced by the simultaneously overexpression of Lro1p and Dga1p (both encoding acyltransferases) prolongs the chronological as well as the replicative lifespan of yeast cells. The increased number of LDs reduces mitochondrial fragmentation as well as mitochondrial ROS production, both phenotypes that are signs of aging. Strains with an altered LD content or morphology as in the *sei1∆* or *lro1∆* mutant lead to a reduced replicative lifespan. In a yeast strain defective for the LON protease Pim1p, which showed an enhanced ROS production, increased doubling time and an altered mitochondrial morphology, a *LRO1* overexpression resulted in a partially reversion of this “premature aging” phenotype.

## Introduction

Lipid droplets, an organelle surrounded by a phospholipid monolayer and filled with triacylglycerols and sterol esters are getting into the focus of researchers as an important element in cellular detoxification (Geltinger et al., 2020a). It was reported that LDs can function as a storage site for lipophilic toxins (Chang et al., 2015; Hammoudeh et al., 2020) as well as drugs (Dubey et al., 2020) and fat-soluble vitamins (e.g. Vitamin A, D, E and K) (Thiam et al., 2013). Furthermore, LDs can reduce lipotoxicity in the cell by absorbing free fatty acids, in particular free saturated palmitate (C16:0) (Plotz et al., 2016). It has to be stated that free fatty acids are toxic as, by acting as a detergent, they can disrupt membranes as well as proteins (Geltinger et al., 2020a). The situation is even more complex, because some monounsaturated fatty acids are under suspicion to be cytoprotective. A typical example is oleate that was reported to be either toxic (Plotz et al., 2017) or life prolonging for cells (Kim et al., 2017). This controversy will be addressed in the current study.

Besides being a reservoir for hydrophobic substances, LDs are also a hub for proteins, especially harmful and damaged ones (Felder et al., 2021). It was shown that in times of ER stress aggregates are formed at this organelle and hence damaged proteins are passed on from the ER to LDs. Finally, these protein decorated LDs are degraded in the vacuole in a process called microlipophagy (Vevea et al., 2015). Furthermore, LDs can assist in dissolving cytosolic inclusion bodies by the disposition of sterols which act as detergents (Moldavski et al., 2015). Recently, we demonstrated that upon stress induction the physical interaction between mitochondria and LDs increases (Bischof et al., 2017). This is in concordance with previous publications that suggested a stress dependent interlinkage of these two organelles (Shaw et al., 2008; Wang, 2013). This increased contact leads to a process, where some harmful proteins are shuttled form mitochondria to LDs. The protein removal from the outer mitochondrial membrane (OMM) increased the general cellular fitness and promoted the resistance of cells against some pro-apoptotic stimuli. This study was performed in yeast cells (*Saccharomyces cerevisiae*) as well as in mammalian cell lines (Bischof et al., 2017). During stress, the number of proteins at LDs is triplicated in yeast cells, whereas the number of proteins at mitochondria are quite stable. In addition, a stress and age dependent change in LD lipid composition was observed (Geltinger et al., 2020b).

During aging mitochondria play a central role and are the interface between life and death. Age dependent changes at mitochondria are manifold and are listed in the following: a fragmentation of the mitochondrial network; a reduced number of mitochondria; increased oxidative damage (lipids as well as proteins); mitochondrial DNA (mtDNA) mutagenesis; an increased premature leakage of electrons to oxygen in the electron transport chain (ETC) resulting in an increased ROS production; a reduced enzymatic activity; a loss of mitochondrial membrane potential and a reduced respiration and thus energy production (for detailed reviews (Breitenbach et al., 2014; Nilsson and Tarnopolsky, 2019).

Although some hints in literature are present (Beas et al., 2020), there is no experimental evidence that LDs can promote lifespan in yeast cells by improving mitochondrial health. In the current study, we can show that a stimulation of cellular LD numbers can increase both, chronological as well as replicative lifespan in yeast cells. Furthermore, we can show that a petite yeast strain with increased protein damage has a reduced replicative lifespan and a strong growth retardation. Both of these phenotypes can be reverted by raising the cellular LD content.

## Materials and Methods

### Yeast Strains

The *S. cerevisiae* BY4741 strain background (MATa his3Δ1 leu2Δ0 met15Δ0 ura3Δ0) was used for all experiments. Deletion mutants were obtained from the EUROSCARF deletion collection. The deletion mutant *pim1∆* was harboring second site mutations, therefore the strain was recreated for this study. The cells were cultivated at 28°C in complex medium (YPD/YPGal (1% (w/v) yeast extract, 2% (w/v) peptone and 2% (w/v) D-glucose/galactose) or synthetic complete glucose/galactose medium (SC-glucose/galactose (2% (w/v) D-glucose/galactose, 0.17% (w/v) yeast nitrogen base without amino acids, 0.5% ammonium sulphate and 10 ml of complete dropout mixture (0.2% Arg, 0.1% His, 0.6% Ile, 0.6% Leu, 0.4% Lys, 0.1% Met, 0.6% Phe, 0.5% Thr, 0.4% Trp, 0.1% Ade, 0.4% Ura, 0.5% Tyr per liter) under constant shaking. Galactose media were prepared for strains with vectors, which are harboring a galactose-promotor (pESC-His). For oleate and olive oil experiments (0.05% oleate (v/v) with Tween-80) synthetic complete media with glucose as carbon source were prepared. Solid media were made by adding 2% (w/v) agar. Selection for plasmids was ensured *via* leaving out the respective amino acid(s). In the Supplementary Table S1 all used and created yeast strains are listed.

### Cloning of *ARE2* and *LRO1* into the Vector pESC-*ARE1*/pESC-*DGA1*

BY4741 genomic DNA was isolated from an overnight grown (3 ml YPD) culture. After washing with H_2_O, the cell pellet was resuspended in 500 µL SCE (1 M sorbitol, 20 mM EDTA, 10 mM Na-citrate, pH 7) and 40 µL zymolyase (10 mg/ml). The suspension was incubated for 60 min at 37°C under constant shaking. Cell lysis was executed via addition of 60 µL 10% SDS for 30 min at 65°C without shaking. By using 200 µL 5 M potassium acetate pH 5–5.5 on ice for 60 min protein precipitation was performed. After centrifugation at 14,000 rpm for 10 min, the supernatant was mixed with 700 µL isopropanol and the DNA was precipitated at −20°C. The suspension was centrifuged at 14,000 rpm for 5 min. After washing with 70% EtOH, the pellet was resuspended in H_2_O. PCR was performed using Phusion High-Fidelity DNA Polymerase (NEB, Ipswich, MA, United States). For *ARE2* the primers fwd1 (gtc aag gag aaa aaa ccc cgg atc cAT GGA CAA GAA GAA GGA TC) and rev1 (aaa tca act tct gtt cca tgt cga cTT AGA ATG TCA AGT ACA ACG TAC) were used, for *LOR1* the primers fwd2 (ctca cta aag ggc ggc cgc aAT GGG CAC ACT GTT TCG AAG) and rev2 (atc ctt gta atc cat cga taT TAC ATT GGG AAG GGC ATC) (capital letters: complementary regions to the gene of interest; lowercase letters: complementary regions to the vector). Gel elution and clean-up was performed via Wizard^®^SV Gel and PCR Clean-Up System (Promega, Mannheim, Germany). The vector pESC-His was linearized using the restriction enzymes BamHI-HF and SalI-HF (NEB; Ipswich, United States). Using Gibson Assembly^®^ Master Mix (NEB; Ipswich; United States) according to the manufacturer’s protocol *ARE2* was cloned into pESC-His *ARE1* (Bischof et al., 2017). Respectively *LRO1* was cloned into the vector pESC-His *DGA1* (Bischof et al., 2017). Constructs were sequenced by Eurofins-MWG-OPERON (Ebersberg, Germany).

### *PIM1* Deletion

Via homologous recombination, the gene *PIM1* was replaced by a nourseothricin resistance cassette that was amplified from the vector pSDS4 (Lettner et al., 2010) using GoTaq DNA Polymerase (Promega, Mannheim, Germany) and the primers *pim1Δ* fwd (TTT TCT TTT GGT TTT CGA GGT GCT TGA ACG AAA AGA TTT GCA AAT AGA) and *pim1Δ rev* (ATA TTT ACA GAA TGT TTA AAC AGG TAT TTA ATC CAT TTA GAT GAA AAG CTG CAG AGG TAA ACC CAG A). After gel elution and clean-up via the Wizard^®^SV Gel and PCR Clean-Up System (Promega, Mannheim, Germany), the strain BY4741 was transformed with the deletion cassette (*Yeast transformation and Genomic Integration* Section) and the genomic integration was selected by growth on YPD plates containing 100 µg/ml nourseothricin. A correct integration was controlled by PCR using the primers Pim1 A (GAG AAG ACA AAA CCA GGT GGT AGA T) and Pim1 D (CTT CTT AGA AAA GAG GCA AAG AGG T).

### Yeast Transformation and Genomic Integration

The strain BY4741 was grown to an optical density (OD_600_) of 0.6–0.8 prior to harvesting. After centrifugation at 3,500 *g* for 3 min the cells were washed with LiAc/TE (100 mM Tris, 10 mM Tris, 1 mM EDTA, pH 8.0) and resuspended in 200 µL LiAc/TE. 50 µL of this cell suspension was mixed with 5 µg plasmid DNA, 10 µg/ml single-stranded salmon sperm DNA and 300 μL LPT (100 mM LiAc, 10 mM Tris, 1 mM EDTA, pH 8.0, 50% PEG 3350). This mixture was incubated at 28°C for 30 min under constant shaking. 40 μL DMSO was added and the cells were heat-shocked for 15 min at 42°C. After mixing with 1 ml sterile H_2_O, the suspension was plated on the respective selective media plates. In case of genomic integration the cells were recovered in YPD medium for 2 h at 28°C under constant shaking prior to plating.

### Elutriation

**FIGURE 1:**
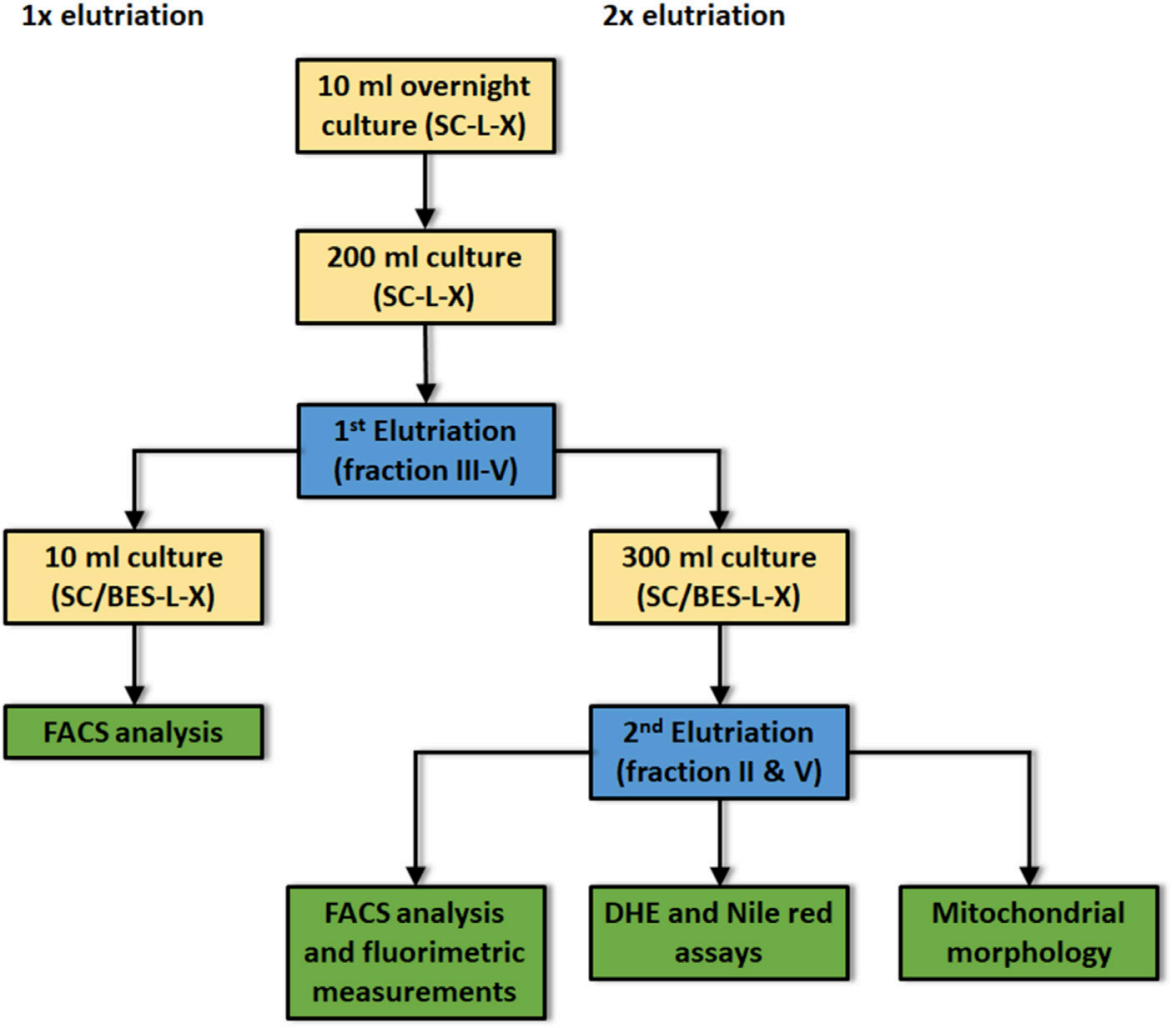
Flow scheme for the isolation and characterization of young and aged yeast cells. Yellow boxes: culture conditions; Blue boxes: either first or second elutriation; Green boxes: Assays performed after first or second elutriation; SC: synthetic complete medium; L: leucine, -L indicates the absence of leucine in the culture medium; X: either histidine or uracil; -X indicates the absence of either his or ura in the culture medium. Before first elutriation cells were cultivated for 24 h at 28°C under constant shaking. Before second elutriation: Cells were cultivated for 48 h at 28°C. The buffer BES was added to the medium to avoid pH dependent side effects.

The elutriation centrifugation was performed as described in Klinger et al. (2010) to obtain a separation of young (is defined as either one or two generations) and old (which is defined as at least fifteen generations) yeast cells according to their replicative age. A workflow of the whole protocol is presented in Figure 1. A 10 ml overnight culture was diluted to an OD_600_ = 0.1 in 200 ml either complex or synthetic medium. After 24 h of growth the first elutriation round was executed. The elutriation was performed with the Beckman elutriation system (Beckman Coulter Inc., Brea CA, United States) and the rotor JE-6B with a standard elutriation chamber (flow rate 10 ml/min). Prior to this process, cells were washed twice with 1xPBS. After resuspension in 10 ml 1xPBS, yeast mother and daughter cells were separated via sonification. Cells were loaded into the elutriation chamber with a rotor speed of 3,500 rpm and a flow rate of 10 ml/min yielding fraction I (virgin cells). Reduction in rotor speed (2,700 rpm) yields fraction II (young cells). Further reduction of the rotor speed to 2,400 rpm yields fraction III and 2000 rpm fraction IV (middle aged cells). Fraction V (old cells) was obtained at a rotor speed of 1,350 rpm. Fraction III, IV and V were reinoculated in 300 ml complex or synthetic medium containing 100 mg/L ampicillin. After 2 days growth at 28°C the second elutriation was conducted to obtain young and old cells. For the FACS analysis main cultures were prepared in SC, SC-Gal, YPD or YP-Gal (OD_600_ 0.1). After incubation for 24 h a first elutriation harboring fraction III-V was conducted. Care was taken that the cells reached stationary phase before elutriation or FACS analysis. Then the cells (OD_600_ 0.1) were further cultivated in 10 ml BES-buffered (100 mM BES at pH 7.5) media containing 100 mg/L ampicillin.

### DHE Staining

2.5 × 10^7^ cells were washed two times with 1x PBS. After centrifugation for 3 min at 3,500 rpm the pellet was resolved in 500 µL PBS-DHE (1:1,000 dilution of a 5 mg/ml DHE stock, Sigma Aldrich—37,291). The samples were incubated for 30 min in the dark without shaking. 200 µL of the solution were pipetted into each well and the fluorescence was measured with Anthos Zenyth 3,100 (Anthos Labtec Instruments GmbH, Salzburg, Austria). Excitation was set at 535 nm and emission was detected at 625 nm for 4 s.

### Nile Red Staining

After washing two times with 1x PBS, 1 × 10^7^ cells in a volume of 225 µL were pipetted into a 96-well plate. The cells suspension was mixed with 25 µL formaldehyde (37%). After addition of 1 µL Nile red (0.001 mg/ml in acetone, Thermo Fisher Scientific, N-1142), the plate was incubated for 20 min in the dark on a shaker and the fluorescence was measured using the Anthos Zenyth 3,100 (Anthos Labtec Instruments GmbH, Salzburg, Austria). Excitation was set at 485 nm and emission was detected at 595 nm for 0.4 s. Because elutriation yields were low cell numbers, a modified protocol was used after elutriation: 0.5 × 10^7^ cells and 0.002 mg/ml Nile red in acetone without formaldehyde fixation were used.

### FACS Analysis

Yeast cells harboring the aging reporter (Supplementary Table S1) were elutriated as indicated in *Elutriation* Section and Figure 1.

After either one or two elutriation rounds the cells were analyzed using the FACS CytoFLEX S (Beckman Coulter, United States) equipped with a laser (excitation wavelength 488 nm) and a GFP filter (emission wavelength 510 nm; 20 nm width) with 100,000 events and a medium flow rate of 30 µL/min. Gating was performed to obtain GFP fluorescence over a certain threshold (above autofluorescence).

### Yeast Chronological Lifespan

Cells were cultured in selective citrate phosphate buffered media [either SC-glucose or SC-galactose, buffered with 64.2 mM Na_2_HPO_4_, 17.9 mM citric acid, pH 6.0 (Wu et al., 2013)]. A typical overnight culture was inoculated in either YPD or SC medium. These cultures were diluted in 100 ml buffered media to an OD_600_ of 0.1. The strains were cultivated over 4 weeks under constant shaking at 28°C. Water loss, after weighing the cultures, was constantly compensated. Survival plating was performed every day. Every week DHE and Nile red stainings were performed. The survival integral (SI), meaning the area beyond the lifespan curves were calculated with online available tools (https://www.desmos.com/calculator/be5ne9vwi8).

### Microscopy

Microscopical analysis was carried out with a Nikon (Tokyo, Japan) Eclipse Ni-U equipped with a DS-Fi2 digital camera, a Nikon Eclipse Ti2 (Tokyo, Japan) and a Leica DMi8 microscope (Wetzlar, Germany).

### Oxygraph Measurements

Overnight cultures (BY4741 pESC-His, BY4741 pESC-*ARE1/ARE2* and BY4741 pESC-*LRO1/DGA1*) were diluted in 2% YPGal (OD_600_ = 0.1) and grown for 48 h at 28°C under constant shaking (600 rpm). Oxygen consumption of 10^8^ cells was measured by using an Oxygraph 2k at 28°C (Oroboros Innsbruck, Austria).

### Statistical Analyses

Data are presented as standard deviations ±SD. Data were tested using one-way ANOVA followed by a TUKEY post hoc test or unpaired two-tailed Student’s t-test, and results with *p* < 0.05 were considered statistically significant.

## Results

### LDs in Aging

**FIGURE 2:**
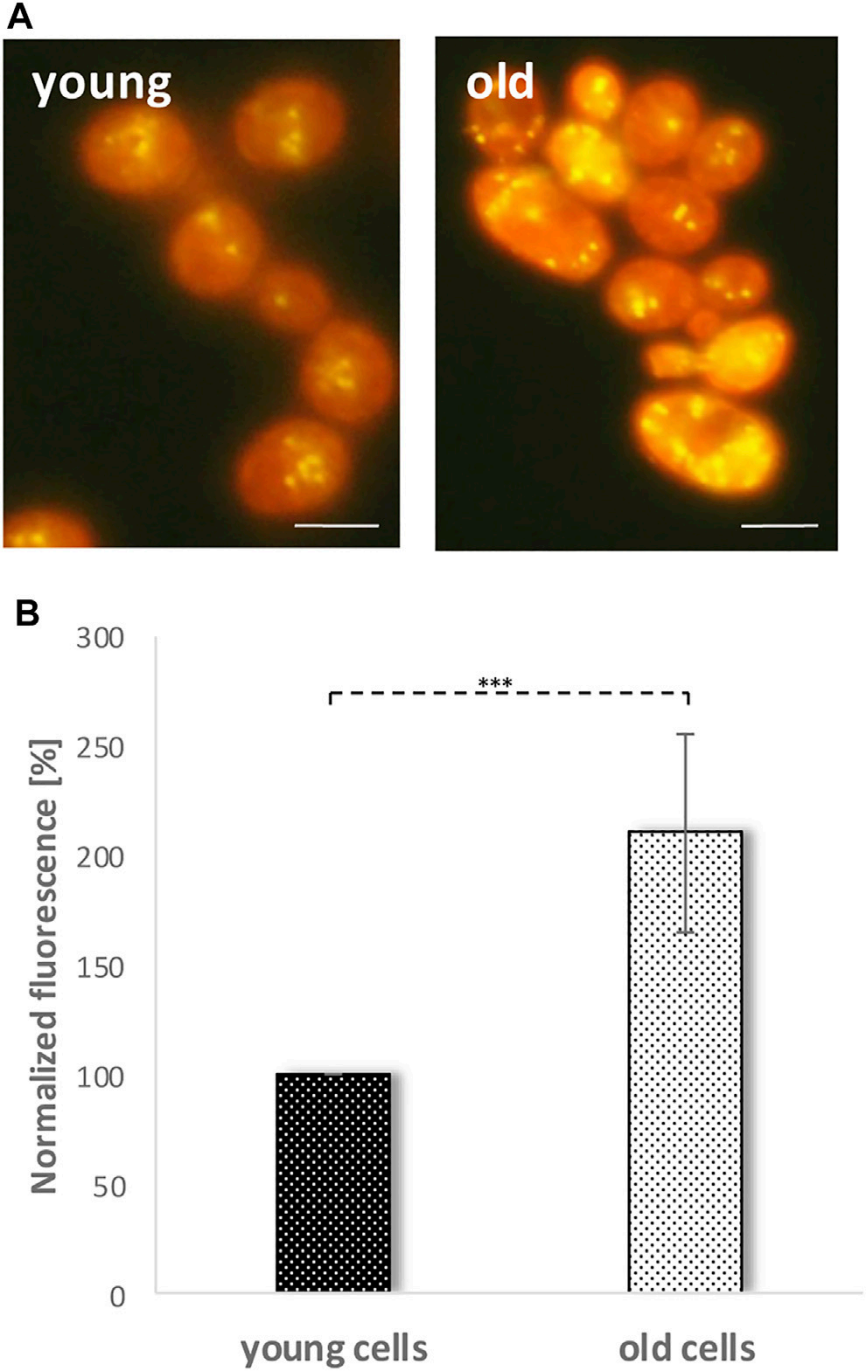
Nile red staining of young and old cells. Fraction II cells (young cells) and fraction V cells (old cells) were obtained by elutriation and were stained with Nile red (0.001 mg/ml). **(A)** LDs are visualized by a yellow-golden fluorescence using a fluorescence microscope. Scale bar: 5 µm. **(B)** An increase in LD numbers and accumulation of neutral lipids was shown *via* fluorometric measurements in a multi-plate reader. Increase of Nile red fluorescence in old cells was calculated *via* value normalizing to fraction II (young cells) fluorescence. (*N* = 7; ***: *p* < 0.01).

Recently, we were able to demonstrate that LDs fulfil an important role in the cellular stress management of *S. cerevisiae* (Bischof et al., 2017). Cells devoid of LDs become sensitive to the application of stressors such as acetic acid or hydrogen peroxide, whereas yeast cells harboring a surplus of LDs show the opposite phenotype. Furthermore, LDs are perfect biomarkers for cellular stress. Immediately after stress induction the LD content increases (Bischof et al., 2017). Based on these findings, we wanted to test the role of LDs in the aging process of yeast cells. In a first approach, replicatively aged cells were isolated via elutriation. This special counterflow centrifugation technique takes advantage of the fact that during aging the cell size as well as the sedimentation coefficient increases (Klinger et al., 2010; Geltinger et al., 2020b). Usually a 300–500 ml cell culture in stationary phase is separated into four fractions (fraction II, III, IV and V). Fraction II represents young and fraction V old cells. Fraction II and fraction V cells were stained with 0.002 mg/ml Nile red. This dye shows a high affinity for neutral lipids and is used as a selective “yellow-gold” or red fluorescent probe for LDs (Greenspan et al., 1985). Fluorescence microscopy clearly revealed a strong accumulation of LDs in aged cells (Figure 2A). This finding was confirmed by fluorometric measurements which showed a two-fold increase in LD content in fraction V compared to fraction II (Figure 2B).

### LDs and Replicative Lifespan

**FIGURE 3:**
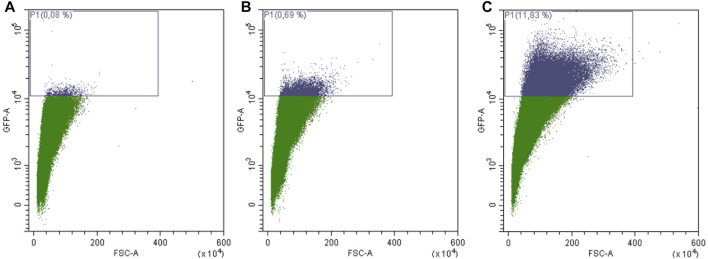
FACS sorting of yeast cells transformed with the aging reporter (YCPlac111-HOprom.-GFP). Young cells/fraction II **(A)**, were separated from middle aged cells/fraction III **(B)** and old cells/fraction V **(C)** by elutriation centrifugation. Single cells were gated based on GFP-A/FSC-A. In **(A)** 0.08% of GFP-high cells were detected, in **(B)** 0.69% of GFP-high cells and in **(C)** 11.83% of GFP high cells can be monitored. The purple rectangle encloses cells with a fluorescence signal above auto-fluorescence level.

The replicative lifespan is based on the observation that yeast cells can perform a limited number of cell divisions. These cell divisions are asymmetrically producing a larger mother cell that ages and a smaller daughter, which rejuvenates itself. The classical way of measuring the replicative lifespan of yeast cells is very tiresome and is dependent on the usage of a micromanipulator to remove and count the constantly produced daughter cells (Mortimer and Johnston, 1959). Therefore we recently established a new method based on an “aging reporter”, which is less labor intensive, faster and allows the screening of several yeast strains in parallel (Streubel et al., 2018). This aging reporter is composed of a cell cycle specific promoter (HO promoter) and the green fluorescent protein GFP. Each time the mother cell divides GFP is expressed. Hence, aged cells show a much brighter fluorescence than younger cells. In this initial publication we performed two subsequent elutriations. The first elutriation is necessary to increase the amount of replicatively aged yeast cells. In this first elutriation fraction II was discarded and fraction III-V were reinoculated and grown for further 2 days (Figure 1). After the second elutriation round aged cells can be isolated in a sufficient amount to perform further studies. To measure the replicative lifespan the increase of fluorescence between fraction II (young cells) and fraction V (old cells) was analyzed using a fluorometer. We showed that both methods, the “classical micromanipulation” and the “aging reporter“, delivered absolutely comparable results concerning some life prolonging interventions [for further details (Streubel et al., 2018)]. In our current study, we further tried to simplify the workflow. In Supplementary Figure S1A FACS analysis data of yeast cells transformed with the vector YCplac111 are presented, whereas in Supplementary Figure S1B cells harboring the aging reporter (YCPlac111-HOprom.-GFP) are shown. As expected cells expressing GFP show a much stronger fluorescence. Afterwards the strain BY4741 YCPlac111-HOprom.-GFP was elutriated two times and fraction II (young cells), fraction III (middle-aged cells) and fraction V (old cells) were analyzed separately using the FACS CytoFLEX S (Beckman Coulter, United States) (Figure 3). A constant age-dependent increase in GFP fluorescence was observed, confirming the functionality of the aging reporter.

Furthermore, it was tested if there is a necessity for elutriation at all. Therefore, cells were elutriated once and fraction III-V were reinoculated for 48 h. These cells were then analyzed directly by FACs without a second elutriation round and were compared to cells that have undergone no elutriation at all (Supplementary Figure S2). It is quite evident that the number of GFP fluorescent and thus aged cells is enormously increased after one elutriation round. All further experiments were then performed with one elutriation (Figure 1).

**FIGURE 4:**
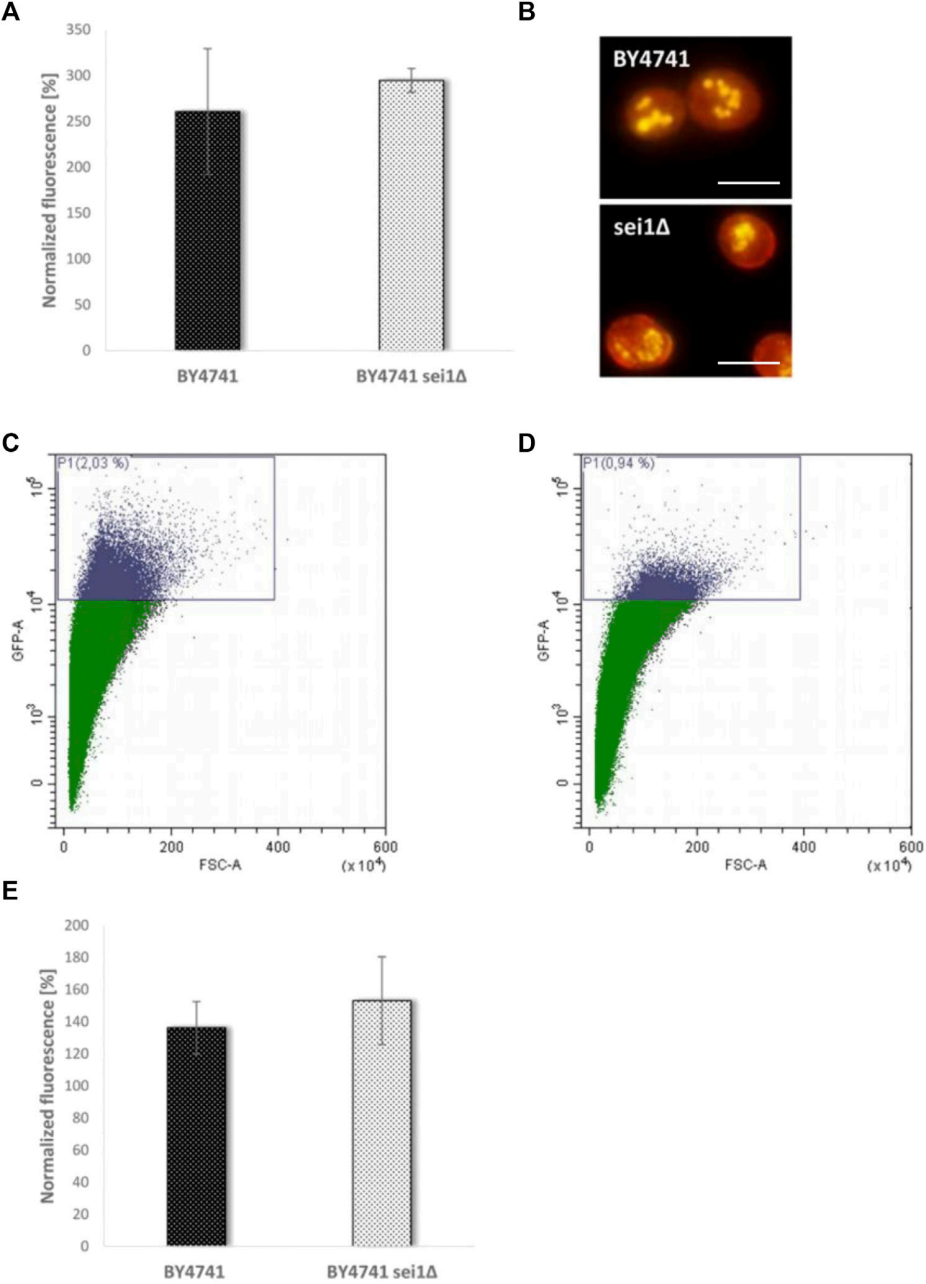
Comparison of the BY4741 and BY4741 *sei1∆* strain. In **(A)** the LD content was determined fluorometrically after Nile Red staining (0.002 mg/ml). The values were normalized to the respective fraction II values (BY4741 fraction V/II, BY4741 sei1∆ fraction V/II). In **(B)** the LD morphology after Nile red staining was studied using a fluorescence microscope. Scale bar: 5 µm. In **(C)** FACS analysis data of the strain BY4741 transformed with the aging reporter (YCPlac111-HOprom.-GFP) are presented (2.03% of GFP-high cells), whereas in **(D)** FACS data of the strain BY4741 *sei1∆* YCPlac111-HOprom.-GFP are shown (0.94% of GFP-high cells). In **(E)** the age dependent increase (comparison of fraction II and V) in ROS levels (DHE staining) is shown for the BY4741 and BY4741 *sei1∆* strain.

To test the role of LDs during mother cell specific aging, we chose a mutant strain that is deficient for the gene *SEI1* encoding the yeast seipin. Seipin is responsible for controlling three parameters of LDs in yeast cells: number of LDs, morphology and size. It is published that in a *sei1∆* mutant strain either supersized or small clustered LDs can be observed (Fei et al., 2008; Wang et al., 2014). Quantification of the LD content (Figure 4A) revealed no significant difference between BY4741 and BY4741 *sei1∆,* but fluorescence microscopy demonstrated differences in LD size and distribution (small and clustered LDs, Figure 4B). After transformation with the aging reporter, elutriation and growth for further 2 days the control strain (BY4741 YCPlac111-HOprom.-GFP) and the deletion mutant (BY4741 *sei1∆* YCPlac111-HOprom.-GFP) were analyzed using the FACS CytoFLEX S. In the deletion mutant strain a clear reduction of cells with a high GFP fluorescence signal was observed (a 1.85-fold decrease of aged cells), indicating a reduction of the replicative lifespan (Table 1; Figures 4C,D). As a second candidate the acyltransferase Lro1p was chosen. This enzyme catalyzes the reaction of diacylglycerols to triacylglycerols. Upon deletion, the LD content decreased and the amount of replicatively aged cells as shown in Table 1 is nearly halved. Because we showed that during stress response a close interaction between mitochondria and LDs exists (Bischof et al., 2017; Geltinger et al., 2020b), this organelle was further analyzed.

**TABLE 1:** Replicative lifespan of several yeast strain. The proportion of aged yeast cells (high GFP fluorescence) was normalized to the respective control strains (either BY4741 p416GPD YCplac111-HO-Prom.-GFP, BY4741 YCplac111-HO-Prom.-GFP, BY4741 YCplac111-HO-Prom.-GFP Tween 80, BY4741 pESC-HIS YCplac111-HO-Prom.-GFP or BY4741 p416GPD pESC-His YCplac111-HO-Prom.-GFP). A value below 100% indicates a reduced replicative lifespan, a value above 100% an increased one. The data were analyzed by an unpaired one-way analysis of variance (ANOVA) followed by a TUKEY post hoc test (*p* < 0.0000). The following comparisons were made: BY4741 YCplac111-HO-Prom.-GFP was compared to strain #1-3. BY4741 YCplac111-HO-Prom.-GFP Tween 80 was compared to strain #4 and #5. BY4741 pESC-HIS YCplac111-HO-Prom.-GFP was compared to strain #6,#7, #10 and #11. BY4741 p416GPD YCplac111-HO-Prom.-GFP was compared to strain #8, #9, #12-15. BY4741 p416GPD pESC-His YCplac111-HO-Prom.-GFP was compared to strain #16.

Strain #	Strain description	Normalized proportion of aged cells	SD	#Biological replicates	*p*-value
1	BY4741 ldb16∆ YCplac111-HO-Prom.-GFP	67.51%	15,96%	3	0.03
2	BY4741 sei1∆ YCplac111-HO-Prom.-GFP	54.04%	10.43%	3	0.00
3	BY4741 lro1∆ YCplac111-HO-Prom.-GFP	56.72%	2.57%	3	0.09
4	BY4741 YCplac111-HO-Prom.-GFP Oleate	77.62%	25.71%	3	0.24
5	BY4741 YCplac111-HO-Prom.-GFP Olive Oil	90.56%	4.36%	3	0.73
6	BY4741 pim1∆ pESC YCplac111 HO-Prom.-GFP	26.34%	9.09%	9	0.00
7	BY4741 pim1∆ pESC-*ARE1/ARE2* YCplac111 HO-Prom.-GFP	73.85%	30.39%	6	0.15
8	BY4741 pim1∆ p416GPD YCplac111 HO-Prom.-GFP	56.35%	7.32%	6	0.18
9	BY4741 pim1∆ p416GPD-*LRO1* YCplac111 HO-Prom.-GFP	202.6%	71.315%	9	0.01
10	BY4741 pESC-*LRO1/DGA1* YCplac111 HO-Prom.-GFP	305.91%	83.45%	8	0.00
11	BY4741 pESC-*ARE1/ARE2* YCplac111 HO-Prom.-GFP	112.11%	22.39%	8	0.90
12	BY4741 p416GPD-*LRO1* YCplac111 HO-Prom.-GFP	160.35%	33.59%	3	0.03
13	BY4741 p416GPD-*DGA1* YCplac111 HO-Prom.-GFP	155.17%	28.089%	3	0.05
14	BY4741 p416GPD-*ARE1* YCplac111 HO-Prom.-GFP	171.11%	11.86%	3	0.01
15	BY4741 p416GPD-*ARE2* YCplac111 HO-Prom.-GFP	210.35%	5.47%	3	0.00
16	BY4741 pESC-*DGA1* p416GPD-*LRO1* YCplac111 HO-Prom.-GFP	203.53%	16.42%	3	0.00

A hallmark of aging is a reduced efficiency of mitochondrial respiration, resulting in a premature leakage of electrons, which are transferred to O_2_ (Lopez-Otin et al., 2013). As a consequence superoxide is produced which can be measured by specific dyes such as dihydroethidium (DHE) (Chen et al., 2013). After elutriation, we compared the DHE levels of fraction II and V. Confirming Harman’s famous observations (Harman, 1956), we saw a clear increase (1.36-fold) in superoxide levels in aged cells. The observed ROS levels further increased in the *SEI1* deletion mutant (1.53-fold), even if this difference was not statistically significant (Figure 4E).

To confirm the previously mentioned findings, ldb16∆ cells were also analyzed. The LDB16 gene encodes a Sei1p interacting protein that is responsible for targeting Seip1p to ER-LD contact sites (Wang et al., 2014). Identical to the BY4741 sei1∆ strain, the ldb16∆ mutant strain shows a strong reduction in replicative lifespan (Table 1).

### LDs Prolong Lifespan

**FIGURE 5:**
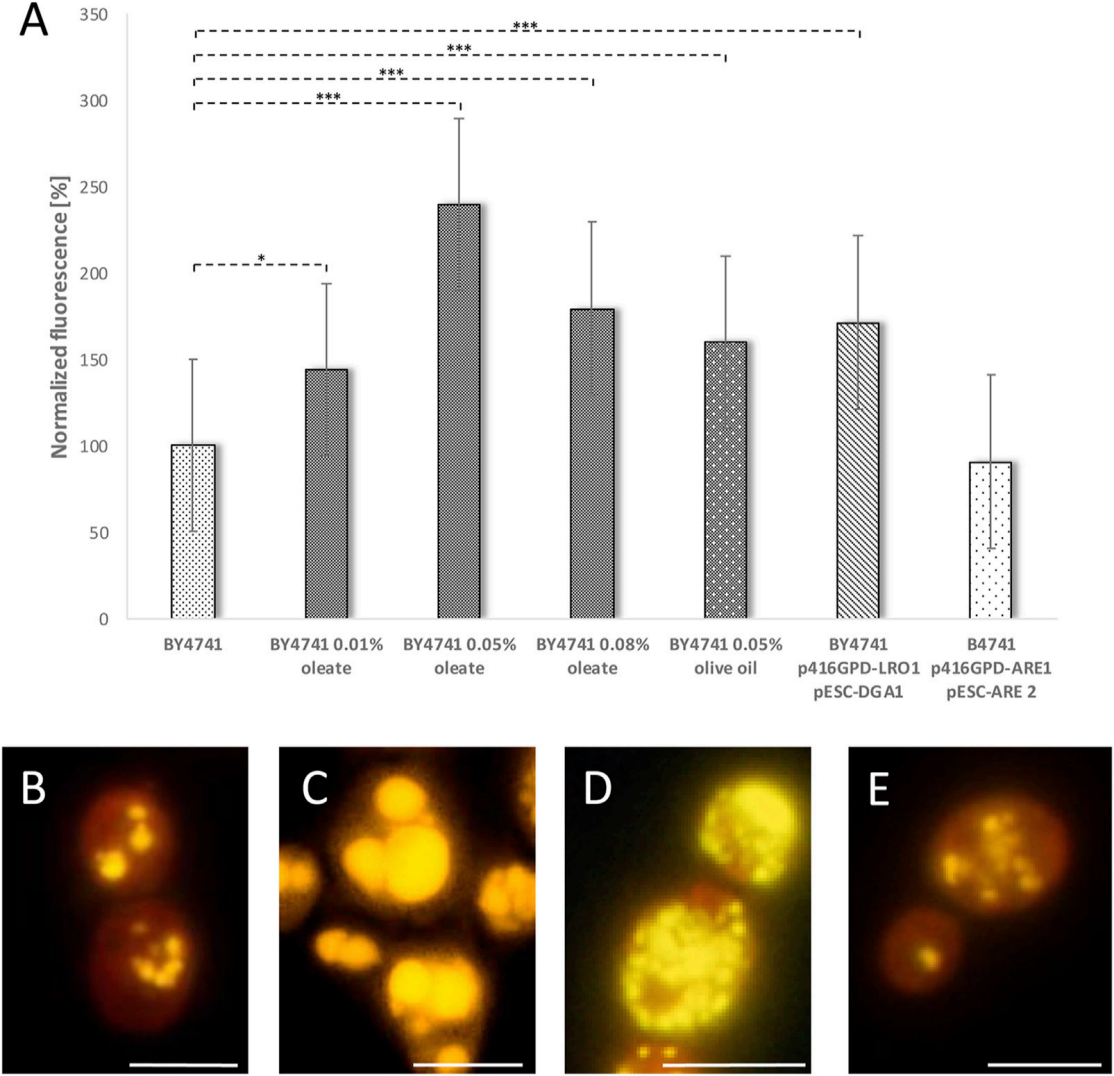
Stimulation of LD content. In the strain BY4741 the LD content was stimulated either by adding 0.01, 0.05 or 0.08% oleate, 0.05% olive oil, *LRO1/DGA1* overexpression (p416GPD-*LRO1*, pESC-*DGA1*) or *ARE1/ARE2* overexpression (p416GPD-*ARE1*, pESC-*ARE2*). LDs were visualized by Nile red staining (0.001 mg/ml). In **(A)** the LD content was measured fluorometrically in a multi-plate reader (Anthos Zenyth 3,100). In **(B–E)** the LD content as well as LD morphology was analyzed by fluorescence microscopy. In **(B)** the strain BY4741, in **(C)** the strain BY4741 supplemented with 0.05% oleate, in **(D)** the strain BY4741 p416GPD-*LRO1* pESC-*DGA1* and in **(E)** the strain BY4741 p416GPD-*ARE1* pESC-*ARE2* is shown. Scale bar: 5 µm.

The findings so far clearly indicate that LDs fulfill a supportive role and the decline in LD numbers or the change in morphology reduces the yeast replicative lifespan. Therefore, we wanted to test, if LDs have the capacity to prolong the lifespan in yeast cells. In a first approach several methods were analyzed which could promote the cellular LD content: Recently we demonstrated that a simultaneously overexpression of DGA1 and LRO1 increases LD numbers. Both genes encode diacylglycerol acyltransferases leading to a surplus of triacylglycerols that are stored in LDs (Bischof et al., 2017). Besides triacylglycerols, LDs also contain sterol esters, which are produced by two Acyl-CoA:sterol acyltransferases (Are1p and Are2p) in yeast cells (Yang et al., 1996). A third way to stimulate LD formation is the addition of the mono-unsaturated fatty acid oleate, the main component of olive oil (Wilfling et al., 2013; Bischof et al., 2017). After Nile red staining all these interventions were analyzed either fluorometrically (Figure 5A) or via fluorescence microscopy (Figures 5B–E). With the exception of an Are1p/Are2p overexpression (BY4741 p416GPD-*ARE1* pESC-*ARE2*) each intervention leads to a significant increase in LD content (Figure 5A). In case of oleate the effect is concentration dependent with a peak at 0.05% oleate. A change in the morphology of LDs was also observed. Addition of 0.05% oleate leads to supersized LDs that completely fill the cell (Figure 5C). In contrast to this finding, a Lro1p/Dga1p overexpression leads to a modest reduction in LD size and to an exploding LD number (Figure 5D). An Are1p/Are2p overexpression had no obvious effect on either LD size or LD number (Figure 5E).

**FIGURE 6:**
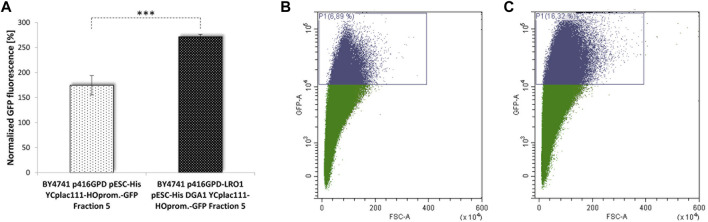
Replicative lifespan in dependence of LDs. Cellular LD content was stimulated by the overexpression of the acyltransferases Lro1p and Dga1p. In **(A)** the GFP signal of the strains BY4741 pESC p416GPD YCplac111-HO-Prom.-GFP and BY4741 pESC-*DGA1* p416GPD-*LRO1* YCplac111-HO-Prom.-GFP was compared fluorometrically after two elutriations (*N* = 4; *p* < 0.001), in **(B)** the GFP signal was analyzed after one elutriation via FACS. In **(B)** 6.89% of GFP-high cells, in **(C)** 16.32% of GFP-high cells were detected.

All these strains and interventions were tested using our aging reporter. After yeast cell transformation with the vector YCPlac111-HOprom.-GFP and a one-time elutriation, the GFP signal was measured via FACS analysis. The strongest GFP signal was obtained for the strain BY4741 p416GPD-*LRO1* pESC-*DGA1.* Compared to the strain BY4741 p416GPD pESC-HIS a more than 2-fold increase in aged cells was observed (Table 1; Figure 6). This result was confirmed by fluorometric measurements. The strains BY4741 p416GPD-*LRO1* pESC-*DGA1* and BY4741 p416GPD pESC-HIS were elutriated twice. The second elutriation yielded young as well as old cells and the fluorescence of these two fractions (II and V) was analyzed separately. Comparing these two fractions the increase in fluorescence (and thus age) is more obvious in the strain BY4741 p416GPD-*LRO1* pESC-*DGA1* than in the control strain (Figure 6A). A further control experiment was performed by using a one-vector system instead of a two-vector system. Both *LR O 1* and *DGA1* were cloned into the vector pESC and both genes were co-expressed from the bidirectional promoter GAL1/10. FACS analysis (Table 1) yielded a more than 3-fold enrichment in aged cells. Both LRO1 and DGA1 were also analyzed separately. Dga1p and Lro1p single-overexpression led to a ∼1.5-fold increase in aged cells (Table 1). This result is clearly indicating that a co-overexpression of Dga1p/Lro1p is life prolonging.

**FIGURE 7:**
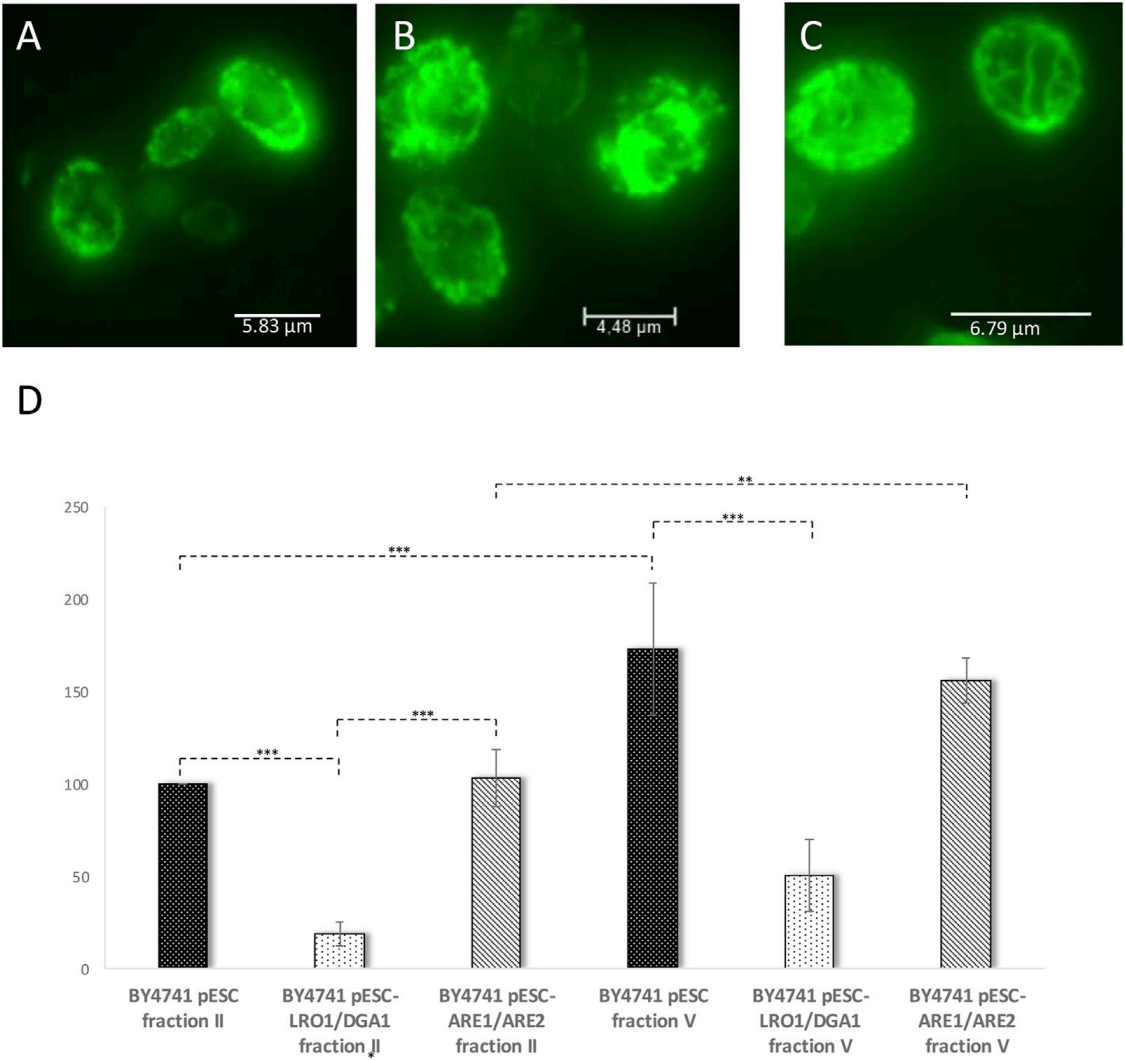
Mitochondrial parameters during replicative aging. In **(A–C)** mitochondria were labelled by transformation with the vector pYX142-mtGFP. The presented images are the result of a maximum intensity projection (z-stack). In **(A)** the mitochondrial morphology of the strain BY4741 pESC was analyzed, in **(B)** the mitochondrial morphology of strain BY4741 pESC-*LRO1/DGA1* and in **(C)** mitochondria of the strain BY4741 pESC-*ARE1/ARE2*. In **(D)** the DHE levels of young (fraction II) and old cells (fraction V) were monitored after elution. The strains BY4741 pESC, BY4741 pESC-*LRO1/DGA1* and BY4741 pESC-*ARE1/ARE2* were used. Statistical analysis was performed using one-way analysis of variance (ANOVA) followed by a TUKEY post hoc test (*p* < 0.0000). In selected cases statistical significance is indicated.

As a co-overexpression of Are1p/Are2p leads to an only modest increase in LD numbers (Figure 5A), the life prolonging effect of this genetic intervention is quite low (Table 1). Only a 1.1-fold non-significant increase in aged-cells was observed. Surprisingly, either an Are1p or Are2p overexpression results in a 1.7-fold or 2.1-fold increase in lifespan, respectively. Non-genetic interventions such as treatment with 0.05% oleate or 0.05% olive oil had no advantageous, but on the contrary detrimental effects (Table 1). This is most probably attributed to the strong morphological changes that LDs have gone through after treatment with these two compounds (Figure 5). Pursuing the life prolonging effect upon LD stimulation, we wanted to test if mitochondria are involved in this process. In healthy cells, mitochondria form a tubular network, whereas in stressed, sick and aged cells, the mitochondrial network starts to fragment (Klinger et al., 2010). In our view, mitochondria are a perfect marker for cellular health. For the visualization of the mitochondrial network cells were transformed with the vector pYX142 harboring GFP fused to a mitochondrial targeting sequence (pYX142 mtGFP) (Westermann and Neupert, 2000). In fact, the mitochondrial network completely collapsed in aged yeast cells that were isolated via elutriation (Figure 7A, Supplementary Movie S1). In cells with boosted LD levels (achieved by either a Lro1p/Dga1p co-overexpression or Are1p/Are2p co-overexpression) no excessive mitochondrial fission during aging was observed [Figure 7B and Figure 7C, Supplementary Movie S2 (Lro1p/Dga1p co-overexpression) and Supplementary Movie S3 (Are1p/Are2p co-overexpression)]. As a second mitochondrial marker, ROS production was monitored. DHE measurements revealed an age dependent increase in superoxide levels in the wildtype strain (BY4741 pESC-His) (Figure 7D). LD stimulation by a Lro1p/Dga1p co-overexpression (BY4741 pESC-*LRO1*/*DGA1*) showed already a significant effect in young cells. In the latter strain the superoxide levels are more than 5-fold decreased (Figure 7D). This finding is even more astonishing when the mitochondrial respiration is taken into consideration. Oxygraph measurements with cells that were grown for 48 h in 2% YPGal were performed. BY4741 pESC-His cells showed a respiration of 28 +/− 4 pmol/(sec*10^7^ cells), BY4741 pESC-*LRO1*/*DGA1* cells a respiration of 61 +/− 13 pmol/(sec*10^7^ cells) and BY4741 pESC-*ARE1*/*ARE2* cells a respiration of 42 +/− 13 pmol/(sec*10^7^cells) (One Way ANOVA; *p* < 0.05).

### Mitochondrial Protein Turnover and Lifespan

**FIGURE 8:**
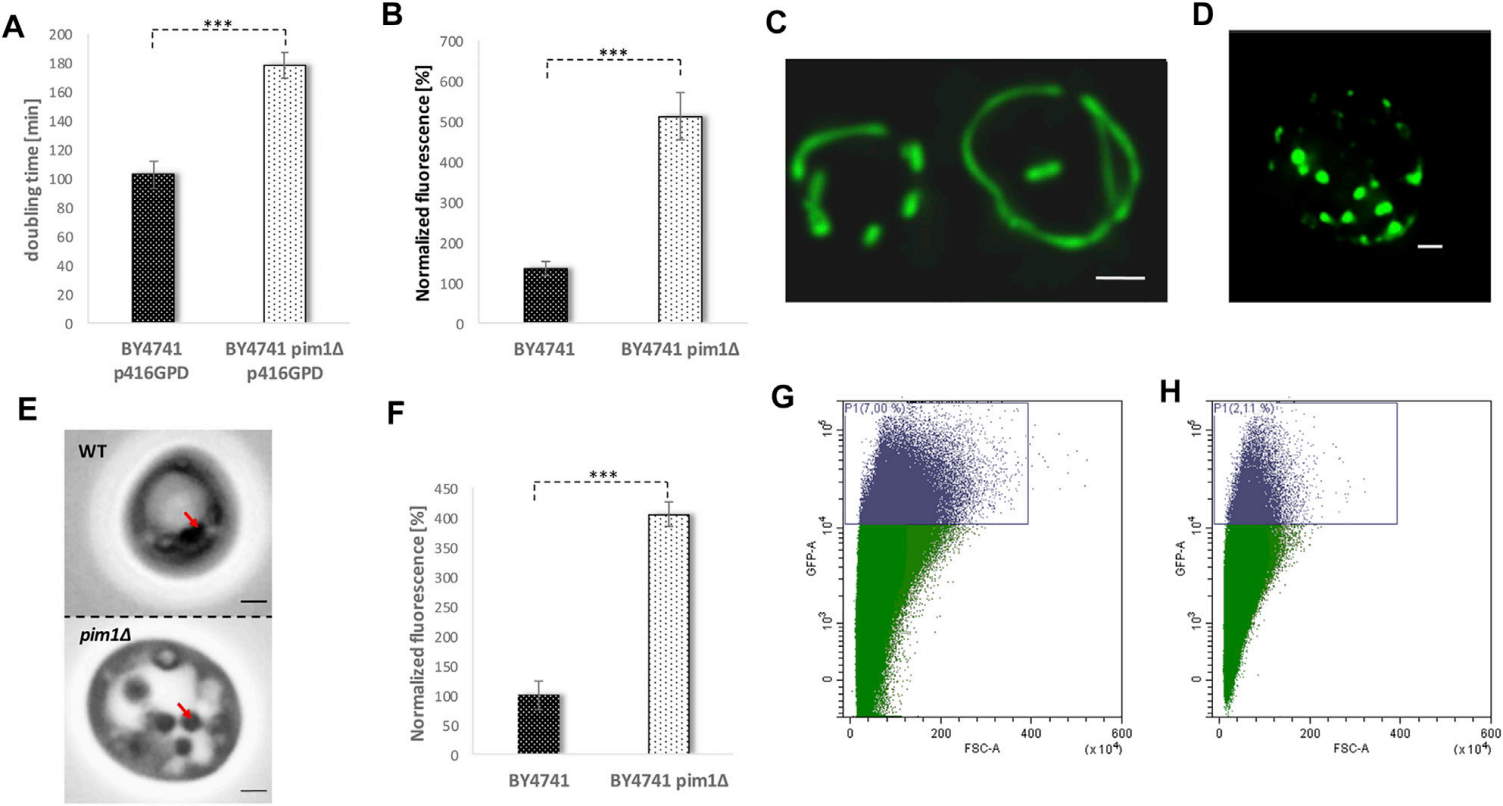
The adverse effects of a *PIM1* deletion. In **(A)** the doubling time during exponential phase of the strain BY4741 p416GPD and BY4741 pim1∆ p416GPD is presented. In **(B)** the ROS levels (superoxide) of the strain BY4741 and BY4741 *pim1∆* were measured using a DHE assay comparing young (fraction II) and aged cells (fraction V). Young and aged cells were separated by two rounds of elutriation centrifugation. After transformation of the strain BY4741 **(C)** and BY4741 *pim1∆* **(D)** with the vector pYX142-mtGFP the mitochondrial morphology was analyzed via fluorescence microscopy. Mitochondria are visualized as a maximum intensity projection (z-stack). Scale bar: 1 µm. In **(E)** DIC images of the strain BY4741 and BY4741 *pim1∆* are shown. Red arrows point to LDs. Scale bar: 1 µm. In **(F)** the LD content was measured fluorometrically after Nile red staining (0.002 mg/ml Nile red in acetone). The strains BY4741 and BY4741 *pim1∆* were analyzed. In **(G)** and **(H)** the replicative lifespan was measured *via* FACS analysis using the aging reporter (YCplac111-HO-Prom.-GFP), which was transformed into the wildtype strain BY4741 **(G)** and the strain BY4741 *pim1∆* **(H)**. In **(G)** 7% of GFP-high cells, in **(H)** 2.11% of GFP-high cells were detected.

There is increasing evidence that in times of stress mitochondria have a central role in protein homeostasis. Protein aggregates that are formed in the cytosol are transported to mitochondria. After import into the mitochondrial matrix, these misfolded proteins are degraded by the LON protease Pim1p. This process was termed *MAGIC* (*m*itochondria *a*s *g*uardian *i*n *c*ytosol) (Ruan et al., 2016). Additionally, we demonstrated that upon stress application and aging LDs get in close contact with mitochondria and a shuttling of proteins from mitochondria to LDs occurs (Bischof et al., 2017; Geltinger et al., 2020b). Most probably, this protein transfer supports *MAGIC*. Therefore, we wanted to test, if the interplay of mitochondrial protein degradation and “LD shuttling” modulates the aging process in yeast cells. In a *PIM1* deletion mutant several mitochondrial parameters are impaired. It has to be stated that the *pim1∆* strain shows no growth on non-fermentable carbon sources (glycerol)/respiratory media, indicative for a petite-like phenotype (Supplementary Figure S3). Consequently, the doubling time in the *pim1∆* strain is increased. The strain BY4741 showed a doubling time of ∼100 min, whereas BY4741 *pim1∆* cells divided every ∼180 min (Figure 8A). After two elutriation rounds, the mitochondrial superoxide production was measured by a DHE-assay comparing fraction II and V. Compared to the wild-type, an enormous age-specific increase in O_2_^−^ levels was observed (Figure 8B). This defect in the *pim1∆* background also manifests on mitochondrial morphology. BY4741 and BY4741 *pim1∆* cells were transformed with the vector pYX142 mtGFP. As visualized by fluorescence microscopy mitochondria form long tubules that fill the mother cells as well as daughter cells (Figure 8C, Supplementary Movie S4). After *PIM1* deletion the mitochondrial network completely collapses and only small, roundish mitochondrial “blobs” remain (Figure 8D, Supplementary Movie S5). This extreme phenotype was observed in 100% of the cells. Compared to the wildtype the GFP signal was very faint in the deletion mutant, indicating a loss of the mitochondrial membrane potential, which is a prerequisite for the mtGFP import. Microscopical investigations revealed another obvious phenotype: The LDs in the *pim1∆* strain were unusually enlarged (Figure 8E). This finding was confirmed by a Nile red staining and fluorometric measurements that showed a 4-fold increase in LDs in the strain defective for the LON protease (8F). Utilizing the vector-based aging reporter, we observed that the GFP signal in the *pim1∆* strain decreases (Figures 8G,H). In dependence of the strain background (either BY4741 pim1∆ p416GPD YCplac111 HO-Prom.-GFP or BY4741 pim1∆ pESC-HIS YCplac111 HO-Prom.-GFP), the amount of aged cells compared to the respective control strains is reduced in the range from 1.8 to 3.8-fold (Table 1).

**FIGURE 9:**
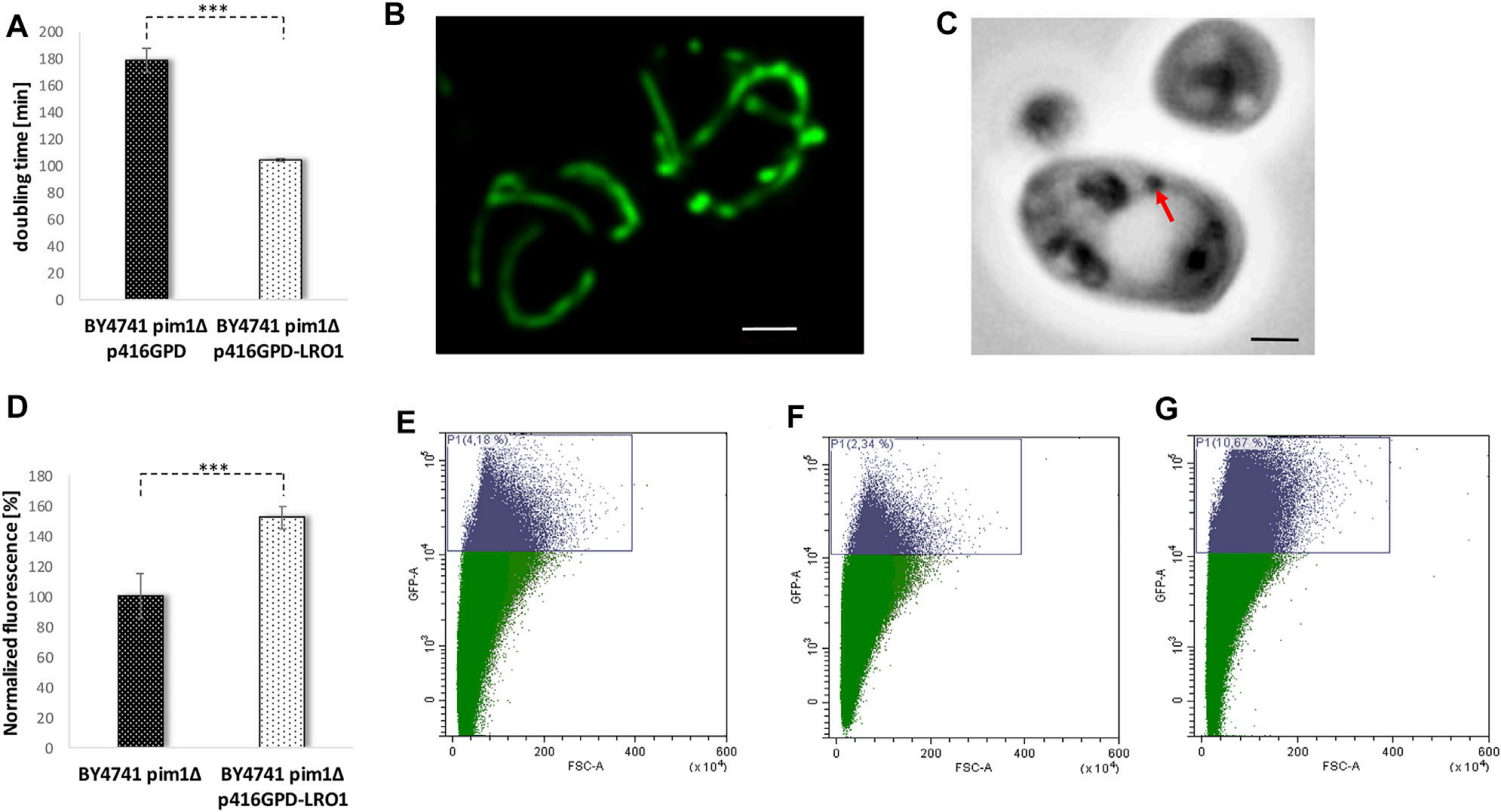
The reversion of the *pim1∆* phenotype. In **(A)** the doubling time of the strains BY4741 *pim1∆* p416GPD and BY4741 *pim1∆* p416GPD-*LRO1* during exponential growth phase was observed. In **(B)** the mitochondrial morphology was studied in the strain BY4741 *pim1∆* p416GPD-*LRO1* pXY142-mtGFP. The presented image is the result of a maximum intensity projection (z-stack). Scale bar: 1 µm. In **(C)** the DIC image of a typical cell of the strain background BY4741 *pim1∆* p416GPD-*LRO1* is shown. Scale bar: 1 µm. In the very same strain the neutral lipid content was measured fluorometrically after Nile red staining **(D)**. In **(E–G)** FACS analysis data (GFP-A/FSC-A plots) of the strains BY4741 p416GPD (E), BY4741 *pim1∆* p416GPD **(F)** and BY4741 *pim1∆* p416GPD-*LRO1* **(G)** are presented. In **(E)** 4.18% of GFP-high cells were detected, in **(F)** 2.34% of GFP-high cells and in **(G)** 10.67% of GFP high cells can be seen.

In further experiments, we tried to reverse some of these premature aging phenotypes by stimulating the LD content. An increase of the LD content by a *LR O 1* overexpression (transformation with the vector p416GPD-*LRO1*) clearly had an enhancing effect on the growth rate of a *pim1∆* strain. The doubling time decreased from ∼180 min in the deletion mutant (BY4741 pim1∆ p416GPD) to 104 min in the strain BY4741 pim1∆ p416GPD-*LRO1* (Figure 9A).

In addition, the mitochondrial morphology was recovered by boosted LD levels. Transformation of cells with the before-mentioned vector induced a complete shift of the fragmented towards a tubular mitochondrial network in the *pim1∆* strain (observed in ∼80% of all cells) (Figure 8D and Figure 9B). As can be seen in Supplementary Movie S6, the recovery was not effective in each single cell, in ∼20% of all cells a partial recovery was observed. Despite the fact that an Lro1p overexpression in the *pim1∆* background further boosted the amount of neutral lipids, a decrease in LD size was observed (Figures 9C,D). In case of replicative aging this phenotype is even more distinct. As can be seen in Figures 9E–G, a *LR O 1* overexpression is not only compensating the aging defect in the *pim1∆* mutant, but is also leading to a prolonged lifespan. The strain BY4741 *pim1*∆ p416GPD-*LRO1* showed a 1.6-fold increase in aged cells compared to the corresponding wildtype and a 2.8-fold increase compared to the *pim1∆* strain (Table 1).

### The Role of LDs in Chronological Aging

**FIGURE 10:**
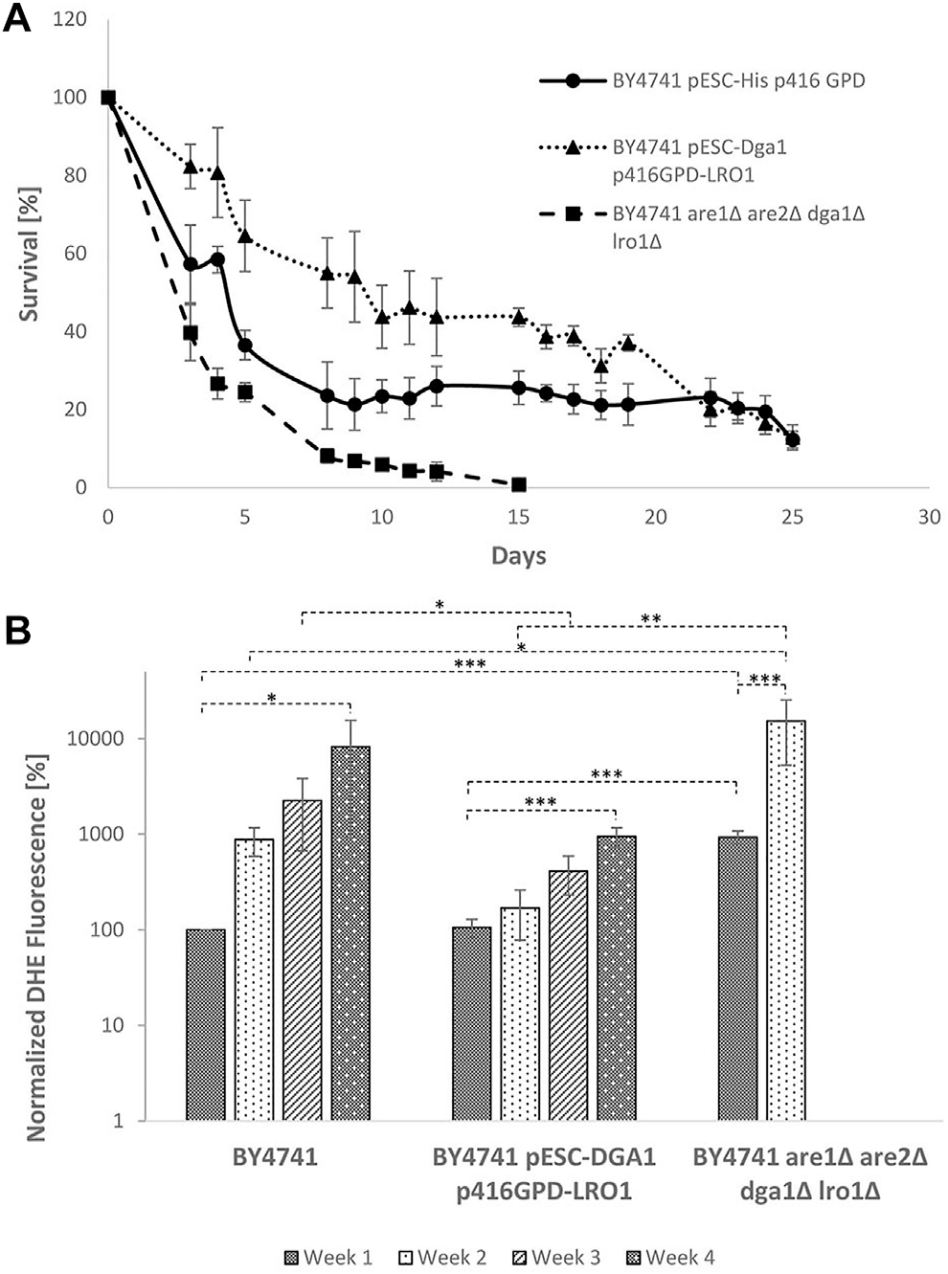
Chronological lifespan in dependence of LDs. **(A)** Survival of the strains BY4741, BY4741 pESC-*DGA1* p416GPD-*LRO1* and BY4741 *are1∆ are2∆ lro1∆ dga1∆* during stationary phase. Data are presented as mean +/− SEM. **(B)** DHE analysis of the strains BY4741, BY4741 pESC-*DGA1* p416GPD-*LRO1* and BY4741 *are1∆ are2∆ lro1∆ dga1* after 1, 8, 15 and 22 days in culture. DHE values were corrected for cell survival and are normalized to the wildtype (first day in culture).

In contrast to the replicative lifespan, chronological aging is focusing on postmitotic cells. This aging process is measured by a loss of viability during stationary phase. In a meta-analysis, we compared the chronological and replicative aging process as well as the underlying pathways and found no significant overlap between these two aging mechanisms (Laun et al., 2006). Nonetheless, the role of LDs during chronological aging was studied. The following yeast strains were cultured for 25 days in buffered SC medium and cell survival was studied using a survival plating assay: BY4741; BY4741 pESC p416GPD; BY4741 pESC p416GPD- *DGA1*; BY4741 pESC p416GPD- *LR O 1*; BY4741 pESC-*DGA1* p416GPD-*LRO1*; and BY4741 *are1∆ are2∆ lro1∆ dga1∆*. Survival curves for selected yeast strains (BY4741 pESC p416GPD; BY4741 pESC-*DGA1* p416GPD-*LRO1*; and BY4741 *are1∆ are2∆ lro1∆ dga1∆*) are presented in Figure 10A, the survival integrals can be found in Supplementary Table S2. The wildtype showed a reduced survival rate after 5 days, the decrease was slowed down and stayed at 20% after 10 days. A strain completely devoid of LDs [BY4741 *are1∆ are2∆ lro1∆ dga1∆* (Bischof et al., 2017)] started to show a sharp decrease in cell survival immediately after the first day, leading to no surviving cells after 15 days in culture. A strain with increased LD numbers (BY4741 pESC-*DGA1* p416GPD-*LRO1*) showed similar death kinetics as the wildtype strain but the survival rate reached a constant level at ∼50% cell survival. From the survival curves quantifiable “survival integrals” (SI) were calculated (Murakami and Kaeberlein, 2009). These SIs confirm prolonged chronological lifespans for all LD stimulation interventions (*LRO1* overexpression; *DGA1* overexpression; and *DGA1/LR O 1* co-overexpression) and clearly demonstrate a loss of viability during stationary phase for the strain BY4741 *are1∆ are2∆ lro1∆ dga1∆* (Supplementary Table S2)*.* In addition to survival plating DHE measurements were performed every week. In case of the wildtype a constant increase in superoxide levels was observed. The same increase was monitored for the strain with the co-overexpression of Lro1p/Dga1p (BY4741 pESC-*DGA1* p416GPD-*LRO1*), although on clearly reduced levels (Figure 10B). A strain devoid of LDs (BY4741 *are1∆ are2∆ lro1∆ dga1∆*) showed a burst of ROS already during the first week. A similar high DHE level was observed in the strain BY4741 pESC-DGA1 p416GPD-*LRO1* after 4 weeks of cultivation.

## Discussion

The aging process in eukaryotes is tightly associated with the increasing appearance of protein aggregates. In yeast cells a broad variety of such aggregates exists, which differ in their cellular localization. In the nucleus the JUxtaNuclear Quality Control Compartment (JUNQ)/IntraNuclear Quality Control Compartment (INQ) (Kaganovich et al., 2008) can be found, the Insoluble PrOtein Deposit (IPOD) forms in close proximity to the vacuole (Kaganovich et al., 2008) and the cytosol is plastered with aggregates such as Q-bodies (Buchan et al., 2008), stress granules (Buchan et al., 2008), heat stress granules (Grousl et al., 2009) and CytoQ/stress foci (Spokoini et al., 2012)/q-bodies (Escusa-Toret et al., 2013) (certain aggregates are eventually identical). Some of these aggregates appear in a stress dependent manner. Stress granules, heat stress granules and Q-bodies can only be observed after a stress application. Even some organelle specific deposition sites for misfolded proteins were identified. In mitochondria harmful proteins are detoxified via storage in the intramitochondrial protein quality control compartment (IMiQ) (Bruderek et al., 2018). These aggregates are a high burden for the cell and would thus limit their lifespan. Due to an asymmetric inheritance the IMiQ and Q-bodies are retained in the aging yeast mother cells, whereas the daughter cells are free from such aggregates (Liu et al., 2010; Spokoini et al., 2012; Bruderek et al., 2018). It was also demonstrated that protein aggregates are formed during replicative aging and limit the replicative lifespan of yeast cells (Saarikangas and Barral, 2015).

In case of protein homeostasis mitochondria are moving more and more into focus. During mitosis protein aggregates are tethered to maternal mitochondria and are thus retained in the aging mother cells (Zhou et al., 2014). We showed that several proteins (among them Mmi1p) are, upon stress application, either translocating to mitochondria (Rinnerthaler et al., 2006) or are stored in stress granules (Rinnerthaler et al., 2013). Recently it was shown that mitochondria assist the cytosolic proteasome in protein degradation, especially under stress conditions. Ruan et al. demonstrated that Mmi1p is released from aggregates by the disaggregase Hsp104p, is imported into the mitochondrial matrix and is finally degraded by the matrix resident LON protease Pim1p (MAGIC) (Ruan et al., 2016). In 2017 we suggested an alternative detoxification route for Mmi1p. Heat, hydrogen peroxide or proteotoxic stress induced a relocalization of Mmi1p from the cytosol to the surface of mitochondria. These stress applications lead to an increase of physical lipid droplet (LD)-mitochondria interactions, Mmi1p is removed from mitochondria and gets finally degraded in the vacuole (Bischof et al., 2017). It has to be mentioned that neither MAGIC nor LD detoxification is specific for Mmi1p, but holds true for many more proteins (Ruan et al., 2016; Geltinger et al., 2020b).

It was observed that in *pim1∆* cells electron dense particles appear in mitochondria visualized by electron microscopy (Suzuki, 1994). This is most probably attributed to the formation of protein aggregates in this strain. As a consequence the mitochondrial morphology is altered (e.g. reduced numbers of cristae), the mtDNA is lost leading to respiratory incompetent cells and the growth speed is reduced (Suzuki et al., 1994; van Dyck et al., 1998). Furthermore, we showed that this strain is suffering from a burst of ROS and an abnormal increase in LD size. Therefore, we tried to dampen some *pim1∆* phenotypes by the stimulation of the cellular LD content. As indicated in the introduction section LDs are not only a hub for lipids, but also for proteins. Previously we demonstrated that upon stress application LDs and mitochondria increase their physical interaction. During this time of increased contact, lipids are exchanged and proteins are transferred from mitochondria to LDs (Bischof et al., 2017; Geltinger et al., 2020b). A strain devoid of LDs showed an abnormal, clumped mitochondrial morphology (Bischof et al., 2017). Transformation of the *pim1∆* strain with a vector harboring the *LRO1* ORF partially recovered some mitochondrial parameters. The clumped mitochondrial morphology in the deletion mutant is reconverted to perfect tubular structures upon LD stimulation. Independently of the *pim1∆* background, increased LD levels are associated with an increase in mitochondrial fitness. During aging, the mitochondrial network starts to fragment, a process that can be stopped by boosted cellular LD levels. This finding is in fact surprising. It is well accepted that LDs are a sort of energy reservoir that fuel mitochondria with lipids for beta-oxidation (Aon et al., 2014). We confirm this observation by showing that cells with increased LD levels (either Are1p/Are2p co-overexpression or Lro1p/Dga1p co-overexpression) have a higher respiratory rate. One would assume that the higher oxygen consummation is accompanied with a higher ROS production, but the contrary is the case. In young as well as aged cells a Lro1p/Dga1p co-overexpression significantly reduces the superoxide levels. This is indicative for “fitter” mitochondria with a reduced premature leakage of electrons to oxygen by the electron transport chain.

Surprisingly, an overexpression of LRO1 decreased the LD size in the *pim1∆* strain. However, this decrease in LD size was accompanied by an increase in LD numbers. An obvious phenotype is the boost of the growth rate in the pim1 deletion mutant after an Lro1p overexpression induced LD stimulation. To sum up, all these data indicate that LDs assist Pim1p in the removal of harmful and misfolded proteins as well as aggregates.

Due to the toxicity of protein aggregates (Stefani and Dobson, 2003) it seems plausible that interventions, which assist their removal, contribute to cellular health and longevity. We clearly demonstrated that gene deletions which reduce the availability of LDs hamper the replicative lifespan in yeast cells. In the *sei1∆* and *ldb16∆* strains, showing an increased association with the ER, a reduction of the replicative lifespan was observed. On the contrary, all interventions that increase the cellular LD numbers (Lro1p overexpression, Dga1p overexpression, Lro1p/Dga1p co-overexpression, Are1 overexpression, Are2p overexpression and Are1p/Are2p co-overexpression) resulted in an enhanced replicative lifespan. This effect cannot be generalized. Treatment of cells with oleate and olive oil led to an accumulation of neutral lipids, which are also stored in LDs. Contrary to genetic interventions mentioned above, a clear change in LD morphology can be seen. The LD numbers decreased and giant-sized organelles started to appear. This phenomenon holds also true for the *pim1∆* background. In contrast to the small, numerous LDs observed upon Lro1p/Dga1p overexpression, this giant sized LDs (after *PIM1* deletion and oleate treatment) limit the replicative lifespan of yeast cells. It can be speculated that LDs need a certain size to get in contact with mitochondria. In fact, an Lro1p overexpression in the pim1∆ background reduces LD size and fully restores the replicative lifespan.

All the effects described above are not specific for replicative aging, but were also observed for the chronological lifespan. During progression of stationary phase a constant increase in superoxide levels monitored by DHE staining was demonstrated. A strain with gene deletions of *ARE1*, *ARE2*, *LRO1* and *DGA1* showed a clear reduction of the chronological lifespan and a burst of ROS already after the first day of cultivation. Similar to replicative aging a stimulation of LD numbers (Lro1p overexpression, Dga1p overexpression and Lro1p/Dga1p co-overexpression) rendered to be very beneficial for the survival during stationary phase. This phenotype was already confirmed by another working group who showed an increase of the chronological lifespan upon Dga1p overexpression (Handee et al., 2016). Especially a strain overexpressing both Lro1p and Dga1p showed a clear reduction of ROS levels in our experiments. This discrepancy increased during survival in stationary phase. With LDs picking up harmful proteins and assisting in the dissolvement of protein aggregates cells can increase their lifespan. In return, LDs contribute to mitochondrial “rejuvenation”, which leads to an enhancement of cellular fitness in the context of chronological and replicative aging.

## Data Availability

The original contributions presented in the study are included in the article/Supplementary Material, further inquiries can be directed to the corresponding author.

## Supplementary Material

Supplementary Figure S1 Aging reporter in yeast cells. In **(A)** a FACS plot of BY4741 YCplac111 cells, in **(B)** the plot of BY4741 cells after transformation with the aging reporter (YCPlac111-HOprom.-GFP) is shown. Single cells were gated based on GFP-A/FSC-A.

Supplementary Figure S2 Comparison of the aging reporter w/wo elutriation. In **(A)** the FACS plot of BY4741 YCPlac111-HOprom.-GFP cells without elutriation are shown, in **(B)** a FACS plot of BY4741 YCPlac111-HOprom.-GFP cells after one elutriation is indicated. Single cells were gated based on GFP-A/FSC-A.

Supplementary Figure S3 Growth of BY4741 and BY4741 *pim1*Δ (several clones) on YPG plates.

Supplementary Movie S1 Mitochondrial morphology of the strain BY4741 pESC pYX142-mtGFP after elutriation (old cells).

Supplementary Movie S2 Mitochondrial morphology of the strain BY4741 pESC-*LRO1/DGA1* pYX142-mtGFP after elutriation (old cells).

Supplementary Movie S3 Mitochondrial morphology of the strain BY4741 pESC-*ARE1/ARE2* pYX142-mtGFP after elutriation (old cells).

Supplementary Movie S4 Mitochondrial morphology of the strain BY4741 pYX142-mtGFP.

Supplementary Movie S5 Mitochondrial morphology of the strain BY4741 *pim1*Δ pYX142-mtGFP.

Supplementary Movie S6 Mitochondrial morphology of the strain BY4741 *pim1*Δ p416GPD-*LRO1* pYX142-mtGFP.
