# Supplementary material for: Lipid Droplets Protect Aging Mitochondria and Thus Promote Lifespan in Yeast Cells
Source: Front Cell Dev Biol. 2021 Nov 19;9:774985. doi: 10.3389/fcell.2021.774985 (PMC8640092; doi:10.3389/fcell.2021.774985)
Supplement: Supplementary file 2 [file Table1.pdf]

Strains	Source
**BY4741**	(Brachmann et al., 1998)
**BY4741 pESC p416GPD**	(Bischof et al., 2017)
**BY4741 pESC p416GPD-*DGA1***	This study
**BY4741 pESC p416GPD-*LRO1***	(Bischof et al., 2017)
**BY4741 pESC-DGA1 p416GPD-*LRO1***	(Bischof et al., 2017)
**BY4741 *are1*Δ *are2*Δ *lro1*Δ *dga1*Δ**	(Bischof et al., 2017)
**BY4741 pESC**	This study
**BY4741 pESC-*ARE1/ARE2***	This study
**BY4741 pESC-*DGA1/LRO1***	This study
**BY4741 YCplac111-HO-Prom.-GFP**	(Streubel et al., 2018)
**BY4741 pESC YCplac111-HO-Prom.-GFP**	(Streubel et al., 2018)
**BY4741 p416GPD YCplac111-HO-Prom.-GFP**	(Streubel et al., 2018)
**BY4741 p416GPD pESC YCplac111-HO-Prom.-GFP**	This study
**BY4741 sei1Δ YCplac111-HO-Prom.-GFP**	This study
**BY4741 pim1Δ YCplac111-HO-Prom.-GFP**	This study
**BY4741 ldb16Δ YCplac111-HO-Prom.-GFP**	This study
**BY4741 pim1Δ pESC YCplac111 HO-Prom.-GFP**	This study
**BY4741 pim1Δ pESC-*ARE1/ARE2* YCplac111 HO-Prom.-GFP**	This study
**BY4741 pim1Δ p416GPD YCplac111 HO-Prom.-GFP**	This study
**BY4741 pim1Δ p416GPD-*LRO1* YCplac111 HO-Prom.-GFP**	This study
**BY4741 pESC-*LRO1/DGA1* YCplac111 HO-Prom.-GFP**	This study
**BY4741 pESC-*ARE1/ARE2* YCplac111 HO-Prom.-GFP**	This study
**BY4741 p416GPD-*LRO1* YCplac111 HO-Prom.-GFP**	This study

**BY4741 p416GPD-*DGA1* YCplac111 HO-Prom.-GFP**	This study
**BY4741 p416GPD-*ARE1* YCplac111 HO-Prom.-GFP**	This study
**BY4741 p416GPD-*ARE2* YCplac111 HO-Prom.-GFP**	This study
**BY4741 pESC-*DGA1* p416GPD-*LRO1* YCplac111 HO-Prom.-GFP**	This study
**BY4741 *sei1*Δ**	Euroscarf
**BY4741 *pim1*Δ**	This study
**BY4741 *pim1*Δ p416GPD**	This study
**BY4741 *pim1*Δ pESC**	This study
**BY4741 *pim1*Δ p416GPD-*Lro1***	This study
**BY4741 *pim1*Δ pESC-*ARE1/ARE2***	This study
**BY4741 pESC pYX142-mtGFP**	This study
**BY4741 pESC-*ARE1/ARE2* pYX142-mtGFP**	This study
**BY4741 pESC-LRO*1/DGA1* pYX142-mtGFP**	This study
**BY4741 pYX142-mtGFP**	This study
**BY4741 *pim1*Δ pYX142-mtGFP**	This study
**BY4741 *pim1*Δ p416GPD pYX142-mtGFP**	This study
**BY4741 *pim1*Δ p416GPD-*LRO1* pYX142-mtGFP**	This study

Supplementary Table 1: Strains used in this study
