# Supplementary material for: Lipid Droplets Protect Aging Mitochondria and Thus Promote Lifespan in Yeast Cells
Source: Front Cell Dev Biol. 2021 Nov 19;9:774985. doi: 10.3389/fcell.2021.774985 (PMC8640092; doi:10.3389/fcell.2021.774985)
Supplement: Supplementary file 4 [file Table2.pdf]

Strain	Mean SI	SEM	p-value
**BY4741**	952	130	-
**BY4741 pESC p416GPD**	746	87	-
**BY4741 pESC p416GPD-*DGA1***	857	95	0.4202
**BY4741 pESC p416GPD- *LRO1***	920	98	0.2305
**BY4741 pESC-DGA1 p416GPD-*LRO1***	1085	138	0.0823
**BY4741 *are1*Δ *are2*Δ *lro1*Δ *dga1*Δ**	339	29	0.0036

Supplementary Table 2: The survival integrals (chronological lifespan) for the yeast strains BY474, BY4741 pESC p416GPD, BY4741 pESC p416GPD-*DGA1*, BY4741 pESC p416GPD- *LRO1*, BY4741 pESC-*DGA1* p416GPD-*LRO1* and BY4741 *are1*Δ *are2*Δ *lro1*Δ *dga1*Δ. Statistical significance was tested via an unpaired one-way analysis of variance (ANOVA) followed by a TUKEY post hoc test.
